# An Analog Circuit Approximation of the Discrete Wavelet Transform for Ultra Low Power Signal Processing in Wearable Sensor Nodes

**DOI:** 10.3390/s151229897

**Published:** 2015-12-17

**Authors:** Alexander J. Casson

**Affiliations:** School of Electrical and Electronic Engineering, The University of Manchester, Manchester M13 9PL, UK; alex.casson@manchester.ac.uk; Tel.: +44-161-306-4801

**Keywords:** discrete wavelet transform, *g_m_*C filters, analog signal processing, low power signal processing

## Abstract

Ultra low power signal processing is an essential part of all sensor nodes, and particularly so in emerging wearable sensors for biomedical applications. Analog signal processing has an important role in these low power, low voltage, low frequency applications, and there is a key drive to decrease the power consumption of existing analog domain signal processing and to map more signal processing approaches into the analog domain. This paper presents an analog domain signal processing circuit which approximates the output of the Discrete Wavelet Transform (DWT) for use in ultra low power wearable sensors. Analog filters are used for the DWT filters and it is demonstrated how these generate analog domain DWT-like information that embeds information from Butterworth and Daubechies maximally flat mother wavelet responses. The Analog DWT is realised in hardware via $g_{m}$C circuits, designed to operate from a 1.3 V coin cell battery, and provide DWT-like signal processing using under 115 nW of power when implemented in a 0.18 μm CMOS process. Practical examples demonstrate the effective use of the new Analog DWT on ECG (electrocardiogram) and EEG (electroencephalogram) signals recorded from humans.

## 1. Introduction

Ultra low power signal processing is an integral part of all modern sensor nodes, and particularly so in emerging wearable electronics for medical applications which need to be easy-to-use, robust and reliably always work [1]. In 2010 authors in the IEEE Signal Processing magazine posed the question: “*What does ultra low power consumption mean?*”; and came to the conclusion that it is where the “*power source lasts longer than the useful life of the product*” [2]. This is exactly what is required for creating truly ubiquitous and wearable sensors. However, to realise such low power signal processing inside the sensor node itself huge advances in power performance are still required.

There is a strong community consensus that the increased use of analog signal processing has an important role in achieving these power advances. For example, [3] summaries the benefits of analog processing as “*analog computation is more energy- and area-efficient at the cost of its limited accuracy, whereas digital computation is more versatile and derives greater benefits from technology scaling*”. This mirrors earlier findings from [2] that “*the old thought that we would be able to virtually eliminate analog and do everything in digital is dying away and we now have the advantage of making tradeoffs between digital and analog implementation for SP* [Signal Processing].” Further examples on the role of analog processing can be found in [2,3,4,5,6,7,8,9,10,11].

Low power embedded signal processing based upon making greater use of the analog domain are thus starting to emerge. [3,11] are both recent papers making use of analog signal processing to power efficiently complement digital signal processing: the rejection of interference signals through feedback loop filtering in [11], and the implementation of core signal processing functions (max, min, multiplication) in [3]. Driven by this there is now a strong emphasis on decreasing the power consumption of existing analog signal processing stages, and in creating new, novel, analog signal processing circuits which can allow greater flexibility in algorithm design, and give more choices for the signal processing approach when working in the analog domain, when creating on-sensor node signal processing.

The Continuous Wavelet Transform (CWT) is a signal processing basis used for providing time–frequency decompositions [12,13] that is an excellent example of signal processing which can be implemented very power efficiently in the analog domain. Several different CWT implementations have been reported in recent years for implementing continuous wavelets without scaling functions (such as the Morlet, Mexican Hat, Gaussian derivatives) using nano-Watts to pico-Watts of power [5,14,15,16,17,18,19,20]. The recent developments in this area started with [14] which introduced a mathematical approximation method, based upon Padé expansions, for mapping a non-causal, ideal, mother wavelet function into the form of a continuous time transfer function that is suitable for circuit realisation. The mathematical approximation method was improved upon in [15] which presented a Gaussian wavelet filter with mathematically optimal dynamic range. Low power operation (down to 45 nW in the case of [15]) is achieved through the use of $g_{m}$C circuit topologies where the power consumption is directly proportional to the centre-frequency of the time–frequency decomposition. [17] and [18] both take a similar approach, using analog domain $g_{m}$C topologies to minimize power. As a result these low power CWT implementations have shown particular utility for the processing of low voltage/low frequency biomedical signals such as from electro-physiology—the ECG (electrocardiogram) from the heart and EEG (electroencephalogram) from the brain. Electro-physiological processing is the focus of [5], [19] and [20]. Based upon this, previous work by the authors of the current paper, [19], demonstrates that CWT analysis of EEG signals can be performed using down to 60 pW of power.

**Figure 1:**
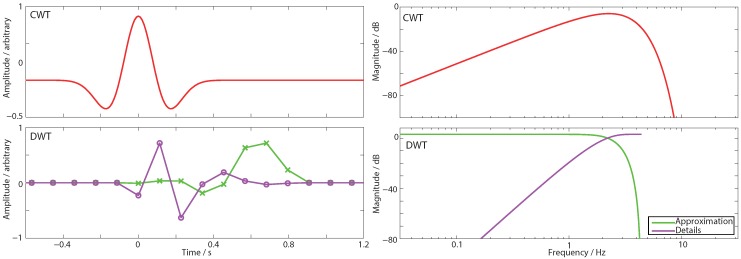
The Continuous Wavelet Transform (**top**), illustrated using the Mexican hat mother wavelet, is a signal processing basis which can be implemented very power efficiently in the analog domain by using fixed *Q* bandpass filters [5,14,15,16,17,18,19]; The Discrete Wavelet Transform (**bottom**), illustrated using the Daubechies 4 mother wavelet, is a related time–frequency decomposition transform which uses separate low pass (approximation) and high pass (details) filters; (**Left**) Mother wavelet time domain impulse response; (**Right**) Corresponding frequency response function.

The Discrete Wavelet Transform (DWT) is closely related to the CWT, Figure 1, and provides alternative time–frequency decompositions. It has proved to be a highly useful signal processing basis and is widely used in applications ranging from data compression, to de-noising, to feature extraction [12,13,21,22]. For example, DWT coefficients have been shown to be one of the single best features for discriminating between seizure and non-seizure sections in the epileptic EEG [23], and is now widely used for the detection of abnormalities in the EEG [24,25]. As a result many VLSI implementations of the 1D DWT have been reported previously [17,26,27,28,29,30,31,32,33,34]. However, until now, only digital domain approaches to the DWT have been considered. The DWT has therefore: not been available as a signal processing choice for novel analog signal processing in sensor nodes; it has not been possible to perform wavelet tree decompositions in analog; and the choice of mother wavelets available for use in analog has been restricted to Morlet, Mexican Hat, and Gaussian derivative CWT wavelets.

This paper presents a new on-chip analog domain approach to approximating the DWT. It is well accepted that the CWT is not restricted to the continuous time domain and that CWT-like information can be generated by discrete time systems; for example using the Matlab cwt function. This paper demonstrates that the converse is true for the DWT: it is not restricted to the discrete time domain, and useful DWT-like information can be generated by analog processing circuits. We therefore provide a new signal processing basis for use in emerging analog signal processing systems, giving designers increased flexibility and algorithm choices when working at very low power levels in wearable sensors. Full circuits for implementing the Analog Discrete Wavelet Transform (ADWT) are presented allowing DWT-like information to be used in on-sensor node analog signal processing systems for the first time.

Section 2 introduces the new ADWT and the time–frequency decomposition provided. This corresponds to a filtering operation with the discrete *z* domain DWT filters mapped to the analog *s* domain, suitable for implementation as continuous time analog filters. These responses are generated for Butterworth and Daubechies maximally flat mother wavelet responses, with the ADWT operation illustrated by decomposing human ECG signals. Section 3 then demonstrates the practical use of the DWT-like information generated by the new ADWT approach by comparing the ADWT and DWT performances when used with an EEG based Artifical Neural Network for determining whether a subject is awake or asleep. Finally, $g_{m}$C circuits realising the ADWT analog filters in a 0.18 μm CMOS process are presented in Section 4. The circuits are designed for the processing of electro-physiological signals in sensor nodes and demonstrate analog domain DWT processing using nano-Watts of power.

## 2. The Analog Discrete Wavelet Transform

### 2.1. Overview

**Figure 2:**
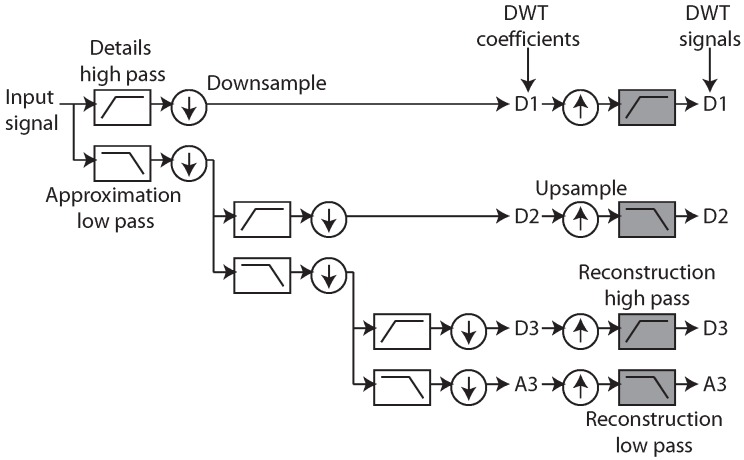
DWT approximation (A) and details (D) *coefficients* are generated via a filter bank and the repeated application of the DWT high and low pass filters. A single stage of the DWT reconstruction process can be used to generate DWT *signals*.

The standard DWT multi-resolution filterbank is illustrated in Figure 2 and acts as the starting architecture for the ADWT. It consists of recursively applied high pass filters for generating *details* and low pass filters for generating *approximations* of the input signal. In the DWT the output of each filter is downsampled by a factor of two before being passed to the next level in the multi-resolution analysis. This process produces DWT coefficients.

The ADWT proposed here operates by generating *z* domain FIR (Finite Impulse Response) filters that implement the forward DWT transform. An estimation procedure (the term *estimation* is used here rather than the more appropriate *approximation* to avoid confusion with the approximation output of the DWT itself) is used to generate *s* domain filters that implement comparable magnitude responses to these *z* domain filters. The ADWT is then performed using the same filter bank approach depicted in Figure 2. In place of the downsampling stage the cut-off frequency of each analog domain filter is scaled by a factor of two. This results in analog domain continuous time signals which define DWT-like *coefficients*. (Note that in general the DWT is invertible and each of the approximation and detail filters is paired with a reconstruction filter. Performing a single stage of the reconstruction process produces the DWT *signals*. Also, by convention, if y[0,...,N] are the DWT coefficients before downsampling, the downsampling process keeps the odd samples in *y*. As a result the DWT is not shift invariant: if the input is delayed by an odd number of points the output is not identical after the delay. The Non-Decimated Wavelet Transform (NDWT), also known as the stationary wavelet transform, eliminates the downsampling stage in order to preserve shift-invariant operation. Apart from this its operation is identical to that of the underlying DWT. Although no equivalent to the decimation stage of the DWT is present in this work, the results show that the ADWT output is a continuous time estimation of the DWT coefficients and not NDWT coefficients, or DWT signals.) In this paper a Butterworth mother wavelet is used due to its potential to estimate the output from Daubechies mother wavelets in the analog domain (Section 2.3) and its suitability for very low power implementation (Section 4).

### 2.2. ADWT Estimation: Magnitude Response

Synthesis of ADWT filters that estimate DWT responses is a matter of finding analog *s* domain transfer functions that match the *z* domain magnitude responses. Methods for mapping digital *z* domain transfer functions to analog *s* domain transfer functions are well established and include the bilinear transform and zero-pole matching [35]. Optimization procedures, such as those commonly used to approximate CWT mother wavelets [15,16], could also be used to select the *s* domain transfer functions that minimise the error between the ADWT and DWT responses. For the ADWT realised here these approaches are not used, instead making use of the close relationship between Daubechies and Butterworth functions.

Both Daubechies wavelets and Butterworth wavelets have maximally flat magnitude responses with frequency, which correspond to them having a maximum number of vanishing moments, and result from all zero and all pole synthesis solutions respectively [36]. Table 1 gives the *z* domain FIR filter coefficients for implementing the Daubechies 1 and Daubechies 4 (dB1, dB4) mother wavelets and this correspondence is easily seen: $1/\sqrt{2}$ factors in the Daubechies 1 equations indicate Butterworth critical damping.

**Table 1:** DWT *z* domain FIR filter coefficients (to three decimal places; A: approximation; D: details).

Mother Wavelet	Filter	Coefficients
dB1	A1	$\;0.707z^{0}+0.707z^{-1}$
	D1	$-0.707z^{0}+0.707z^{-1}$
dB4	A4	$-0.011z^{0}+0.033z^{-1}+0.031z^{-2}-0.187z^{-3}-0.028z^{-4}+0.631z^{-5}+0.715z^{-6}+0.230z^{-7}$
	D4	$-0.230z^{0}+0.715z^{-1}-0.631z^{-2}-0.0280z^{-3}+0.187z^{-4}+0.031z^{-5}-0.033z^{-6}-0.011z^{-7}$

**Figure 3:**
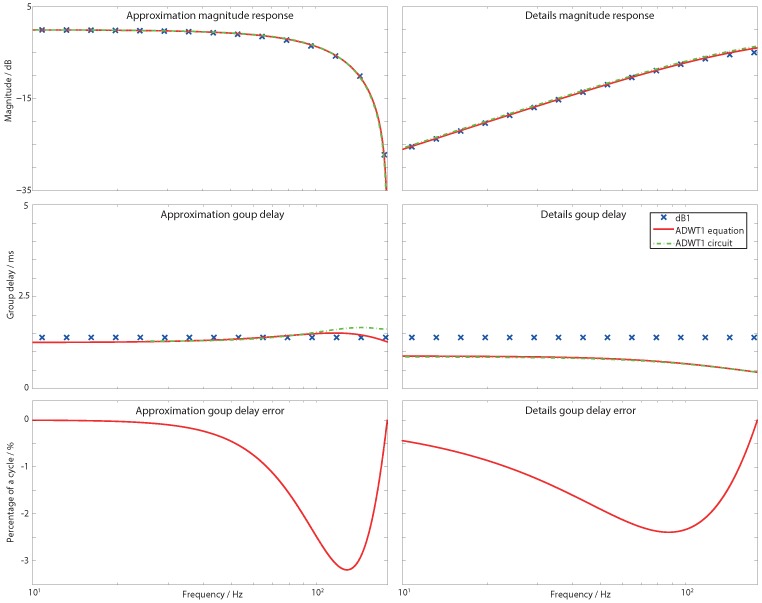
Magnitude, group delay, and group delay error after fixed delay compensation, responses for the DWT and ADWT with base sampling frequency 360 Hz. First order filter Daubechies 1 mother wavelet is shown with passband gain normalized to 0 dB.

Thus the dB1 details (high pass) filter response is flat, with 20 dB per decade roll-off, and is well fitted by a first order Butterworth high pass filter with the cut-off set at half of the DWT base sampling frequency ($f_{s}/2$). Similarly, the magnitude response of the dB1 approximation (low pass) filter is the lower half of a Butterworth notch function. Here $f_{n}=f_{s}/2$, and $Q=1/\sqrt{2}$. The responses for these DWT filters are shown in Figure 3 where an excellent match with the wanted magnitude response is seen. The numerical transfer functions for a DWT base sampling frequency of 360 Hz are given in Table 2.

**Figure 4:**
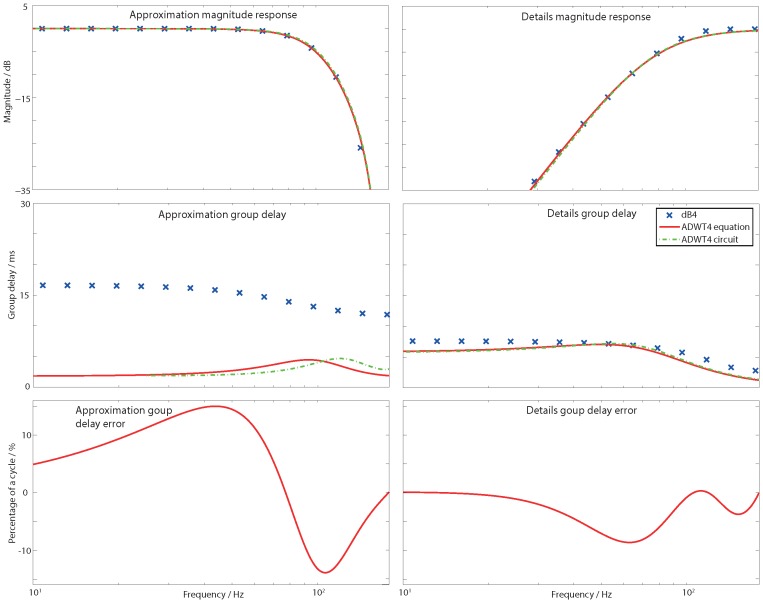
Magnitude, group delay, and group delay error after fixed delay compensation, responses for the DWT and ADWT with base sampling frequency 360 Hz. Fourth order filter Daubechies 4 mother wavelet is shown with passband gain normalized to 0 dB.

The same estimations apply for the magnitude responses of the fourth order dB4 mother wavelet which has an 80 dB per decade fourth order roll-off. The details filter magnitude response is estimated by a fourth order Butterworth high pass filter ($f_{c}=f_{s}/2.34$) while the approximation response is matched by a fourth order notch with $f_{n}=f_{s}/2.12$, Q=0.735. These ADWT responses are shown in Figure 4 with numerical transfer functions given in Table 2. Again these are given for a DWT base sampling frequency of 360 Hz. To perform a multi-resolution ADWT analysis these filters are arranged as a filter bank (Figure 2) and the transfer functions in Table 2 scaled as

(1) $$s\to s/2^{i-1}$$

where *i* is the analysis level.

**Table 2:** Analog transfer functions to match DWT wavelets from Table 1 with base sampling frequency 360 Hz.

Filter	s Domain Transfer Function
A1	$\frac{1.414s^{2}+1.809\times 10^{6}}{s^{2}+1599s+1.279\times 10^{6}}$
D1	$\frac{-9s}{4s+4524}$
A4	$\frac{3.056\times 10^{4}s^{4}+6.973\times 10^{10}s^{2}+3.978\times 10^{16}}{21609s^{4}+4.441\times 10^{7}s^{3}+9.495\times 10^{10}s^{2}+5.067\times 10^{13}s+2.813\times 10^{16}}$
D4	$\frac{-3.65\times 10^{-11}s^{4}}{1.825\times 10^{-11}s^{4}+2.498\times 10^{-8}s^{3}+1.709\times 10^{-5}s^{2}+0.005846s+1}$

### 2.3. ADWT Estimation: Group Delay Response

The ADWT transfer functions in Table 2 define a Butterworth mother wavelet time–frequency decomposition, which also estimates the magnitude response of dB1 and dB4 mother wavelets. They do not correctly estimate the group delays of the Daubechies wavelets however. The group delay error due to the estimation is quantified in Figure 3 and Figure 4 once a fixed delay has been applied to bulk equalize the two transforms. The remaining group delay error is larger when estimating the dB4 wavelet, peaking at 15% of a cycle, with the errors when estimating the dB1 being less than 4%.

**Figure 5:**
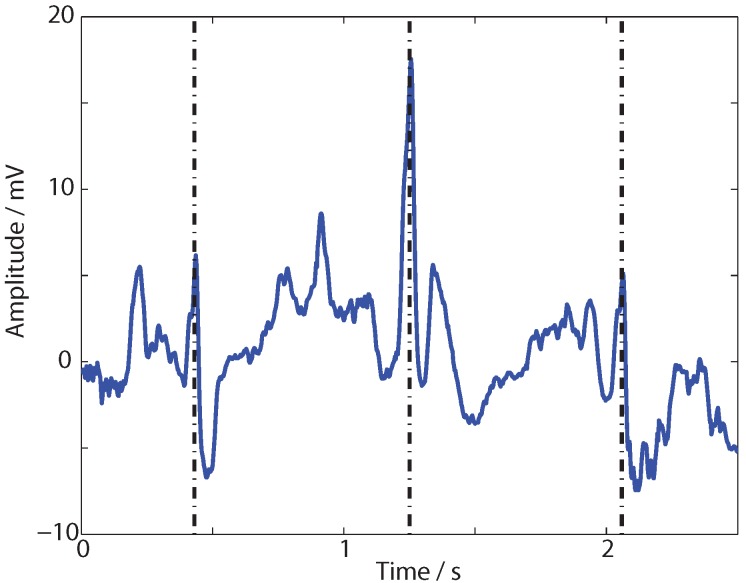
Input noise corrupted ECG signal from Physionet used for testing the ADWT. Black vertical lines indicate heart beats.

**Figure 6:**
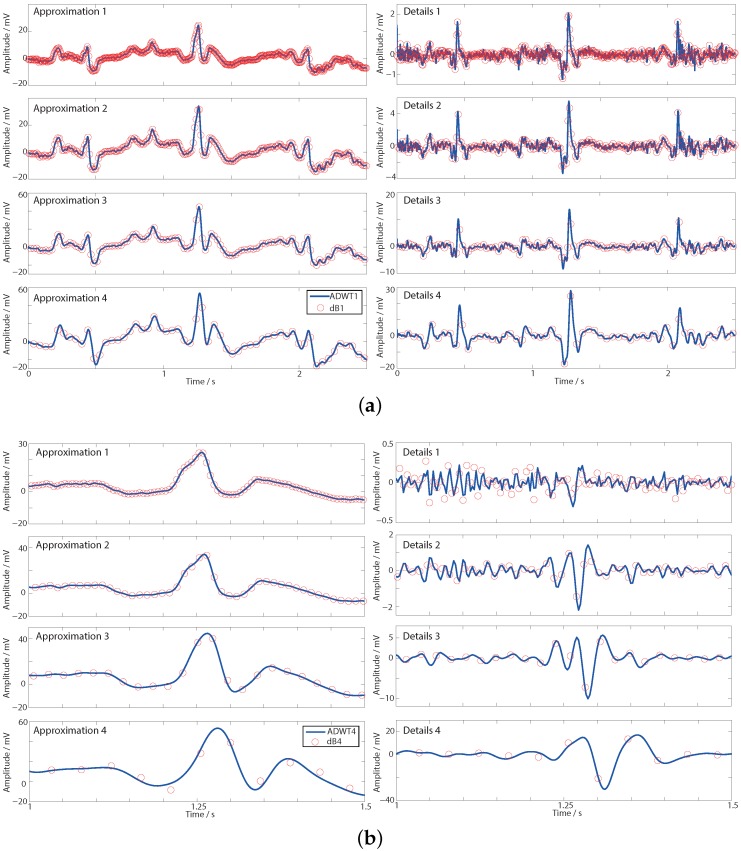
Transient responses of the DWT and ADWT show that the DWT is sampling different points along the underlying ADWT continuous time output. (**a**) First order ADWT1 and dB1; (**b**) Fourth order ADWT4 and dB4.

In this paper, the impact of this remaining group delay error is assessed via a practical example, performing the multi-resolution analysis of a noise corrupted ECG signal taken from Physionet [37,38]. A 2.5 s segment of this signal is shown in Figure 5 in which three heart beats are present, although noise corruption means they are not clearly seen in the time domain. This trace is decomposed using the dB1 and dB4 DWT and the ADWT estimations. The DWT is carried out using the Matlab dwt function and the ADWT using the lsim function with the transfer functions from Table 2. The four level multi-resolution analysis is shown in Figure 6 where a fixed delay has been added to each filter output to align it with the ideal DWT coefficients.

Figure 6a shows the first order, ADWT1 and dB1, responses and it is seen how the DWT details coefficients clearly highlight the heart beats, even though they are not readily apparent in the time domain. Further it is seen that the discrete dB1 output is sampling points on the underlying ADWT1 line produced by the *s* domain ADWT filters. The ADWT output thus contains the intrinsic DWT information from the Daubechies wavelet. In Section 3 this analog domain DWT-like information from the ADWT is taken as the signal processing basis to demonstrate its utility in place of DWT coefficients. Alternatively, if desired, a standard digital DWT could be generated by appropriately sampling the ADWT output. Although the ADWT filters do not match the phase and group delay responses of the ideal FIR DWT filters, an estimation of the DWT filter outputs is generated with only fixed delays necessary to correctly align the coefficients.

Figure 6b shows the same applies for the fourth order ADWT4 and dB4, where for clarity only 0.5 s of data is shown. For most of the approximation and details outputs a similar pattern to Figure 6a is seen with all of the dB4 DWT points falling on the underlying ADWT4 line. However, the ADWT4 level 1 details output is a poor estimation of the dB4 level 1 details. This is because high frequency noise in the input signal is allowed directly through the details high pass filter (no low pass filtering is applied to the input prior to the generation of the level 1 details, Figure 2). As such the minor differences in the filter transfer function estimate have a comparatively large effect on the amplitude and shape of the noise that is passed through. This effect is removed by level 2 once inherent bandlimiting has been imposed by the DWT approximation filter. As the vast majority of DWT decompositions are multi-level this is not a significant limitation of the ADWT estimation for performing signal analysis. Overall, as before direct dB4 DWT information can be generated with appropriate sampling of this ADWT4 line. Alternatively, and as considered below, further signal processing can use the raw DWT-like output of the ADWT4 itself.

### 2.4. Reconstruction

The ADWT implemented here is intended for on-sensor-node signal analyses, for example driving embedded machine learning as considered in Section 3. As such only signal decompositions are required, and detailed consideration of signal reconstruction from ADWT coefficients is beyond the scope of the current sensor node work. Nevertheless, if desired, the inverse filtering based approach introduced in [39] could be used to perform ADWT signal reconstructions to provide an approach towards a full forward and inverse ADWT.

## 3. Application Performance

The utility of the ADWT is demonstrated here by using it in an Artificial Neural Network (ANN) that processes human scalp EEG signals. Two channels of EEG information are used for the two class classification problem of determining whether a subject is asleep or awake. Four, full-day ambulatory recordings of EEG channels FPz–Cz and Pz–Oz sampled at 100 Hz are used [38,40].

The ANN is a five layer feed-forward network [41] (patternnet) which classifies the subject state based upon the DWT generated spectral powers. A four level DWT decomposition is used generating power information in the ranges:

- Details, level 2: 12.5–25 Hz.
- Details, level 3: 6.25–12.5 Hz.
- Details, level 4: 3.125–6.25 Hz.
- Approximations, level 4: 0–3.125 Hz.

This gives a total of eight features for the network to use for classification. The level 1 details coefficients have been excluded from the analysis in-line with the results from Figure 6b (Section 2.3) which showed that the level 1 details output is a relatively poor estimation due to high frequency noise being present directly at the input of the level 1 filter. Physiologically, this corresponds to excluding components from the EEG *gamma band*, those above 25 Hz, from the analysis. This is not an uncommon choice for EEG analysis: while gamma band information has shown utility for sleep analysis [42], the physiological *vs.* artifactual origin of the higher frequency components has been debated [43].

The band powers are generated in 30 s non-overlapping epochs and a classification decision made for each of the 11,318 epochs present over the four EEG recordings. A scaled conjugate gradient backpropagation training routine is used, combined with a leave-one-out cross-validation procedure where the network is trained on three of the records and tested on the fourth. All four permutations are analysed and the reported performance taken as the average of the four out-of-sample tests.

To illustrate the classification performance the two metrics of interest are the (TP: True Positives; FN: False Negatives; FP: False Positives. See [44].):

(2) $$\mathrm{Sensitivity}=\frac{TP}{TP+FN}\times 100\%$$

which gives the fraction of expert marked wake(sleep) epochs that are correctly classified as wake(sleep), and the

(3) $$\mathrm{Selectivity}=\frac{TP}{TP+FP}\times 100\%$$

which shows the fraction of classifications of a given class which are correct. In both cases higher numbers represent better performance and in general there is a trade-off between correctly classifying epochs (having a high sensitivity), and having few false detections (high selectivity).

Results are shown in Table 3 for two different networks. One uses power estimates generated with the dB1 mother wavelet as input features while the other uses dB4 based features. It can be seen how good classification performance is obtained in all cases. The dB1 network achieves higher sensitivities for correctly identifying sleep in the EEG, while the dB4 network achieves higher sensitivities for the detection of wake. There is a similar trade-off in the selectivity with the dB1 network achieving fewer false positives in the wake class and the dB4 network achieving fewer false positives in the sleep class.

**Table 3:** Performance of an Artificial Neural Network for determining wake/sleep state from the EEG using DWT spectral powers.

Training Features;	Wake		Sleep	
Test Features	Sensitivity / %	Selectivity / %	Sensitivity / %	Selectivity / %
	dB1			
DWT; DWT	95.8	98.5	97.2	92.4
DWT; ADWT	95.2	98.7	97.6	91.5
	dB4			
DWT ; DWT	98.1	94.7	89.0	96.0
DWT ; ADWT	98.0	94.2	87.7	96.0

To assess the ADWT, results are also presented in Table 3 for the case when the networks are tested using ADWT generated band powers as the input features. The same artificial neural networks as before are used, trained on the same DWT band powers, only the inputs during the out-of-sample tests have been changed to use the ADWT. In all cases for these networks the classification performances are very similar, and the maximum performance difference is only 1.3%.

Inevitably, the ADWT is an estimation of the underlying DWT and so small differences are present in the ANN output due to the differences in group delay. These are to be expected and their impact will vary between different applications. Nevertheless, the analysis of ECG data in Figure 6 and of 94 h of EEG data in Table 3 has demonstrated the potential use of the ADWT in generating useful DWT-like information. In particular, during deployment of the EEG artificial neural network used here the ADWT based powers could be used in place of the DWT and the classification performance essentially unchanged.

## 4. Fully Custom Circuit Implementation

### 4.1. Design

The objective of the ADWT is to allow the low power on-sensor node generation of analog domain DWT-like information. The functions in Table 2 are generalised *s* domain transfer functions which could be realised using many different circuit techniques and topologies. In order to achieve the lowest possible levels of power consumption this section considers the circuit realisation of the ADWT continuous time notch and high pass filters using fully custom (ASIC) circuits. Here an approach for use in low power, low voltage, low frequency electro-physiological sensor nodes is considered to highlight the low power signal processing utility of the ADWT.

**Figure 7:**
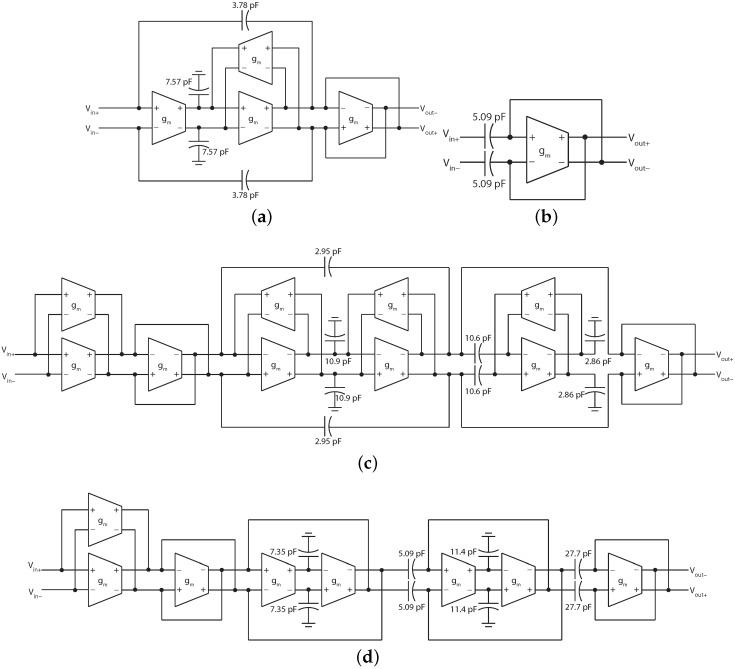
$g_{m}$C circuit topologies realising the ADWT. (**a**) ADWT1 approximation low pass filter; (**b**) ADWT1 details high pass filter; (**c**) ADWT4 approximation low pass filter; (**d**) ADWT4 details high pass filter. All transconductors are the same (Figure 8) with value 12.5 nS.

To achieve low power in this situation $g_{m}$C filter structures are used as their power consumption is directly proportional to the cut-off frequency. This allows very low power consumptions to be achieved when dealing with physiological signals which are themselves very low frequency (typically 1–1000 Hz). They also allow easy cut-off frequency tuning and the ADWT multi-resolution filter bank can be made by using the same filter structure provided with different bias currents. Fully differential topologies realising the four required filters are shown in Figure 7 where all of the filters have a 0 dB passband gain.

**Figure 8:**
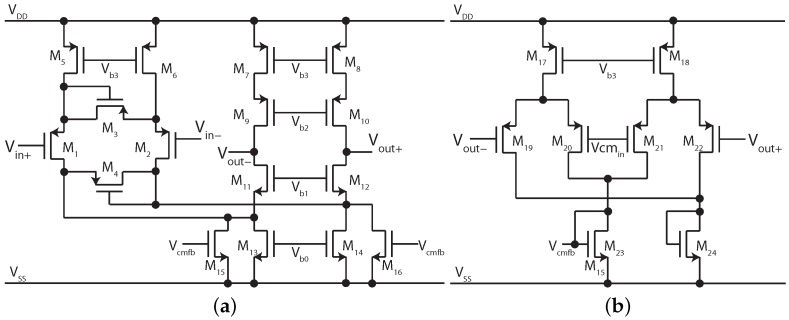
12.5 nS transconductor used in Figure 7. A bias network (not shown) generates voltages $V_{b0}$–$V_{b3}$ from a single input current.

The reported capacitor values are for use with a transconductance of 12.5 nS, readily achievable on-chip using a number of techniques [45]. The transconductor used here is shown in Figure 8 and is based upon a folded cascode with input cross-coupling for transconductance reduction [46]. Further, to achieve very low transconductance the cell is based upon the use of very low currents and the deep weak inversion operating region [19]. Here a nominal bias current of 512 pA is used to give simultaneous low transconductance and low power operation. The cell operates from a 1.3 V supply in order to be directly driven by a single coin cell battery [47] and full transistor sizes are given in Table 4. These are for a triple well, 0.18 μm CMOS process with MIM capacitors, and suitable sizing is a trade-off between the bandwidth, matching and noise [19].

**Table 4:** Transistor sizes from Figure 8. All transistors are high threshold devices, apart from M19–M22.

Transistor	W/L/μm
M1, M2	12/1
M3, M4	4/1
M5, M6	25/5
M7–M10, M19–M22	30/5
M11, M12	14/20
M13, M14	7.1/20
M15, M16, M23, M24	2.9/20
M17, M18	16/5

### 4.2. Simulation Results

The ADWT circuits have been laid out and simulated and the circuit performance is summarised in Table 5. A 512 pA nominal bias current is used to create ADWT filters that match a DWT with a 360 Hz base sampling frequency. Figure 3 and Figure 4 show the implemented magnitude and group delay responses, which closely match both the wanted DWT responses and the mathematical ADWT equations (Table 2). The maximum power consumption of a single filter is 36 nW, and operation is possible over the 2 to 720 Hz range covering the full spectrum of human electro-physiological signals.

**Figure 9:**
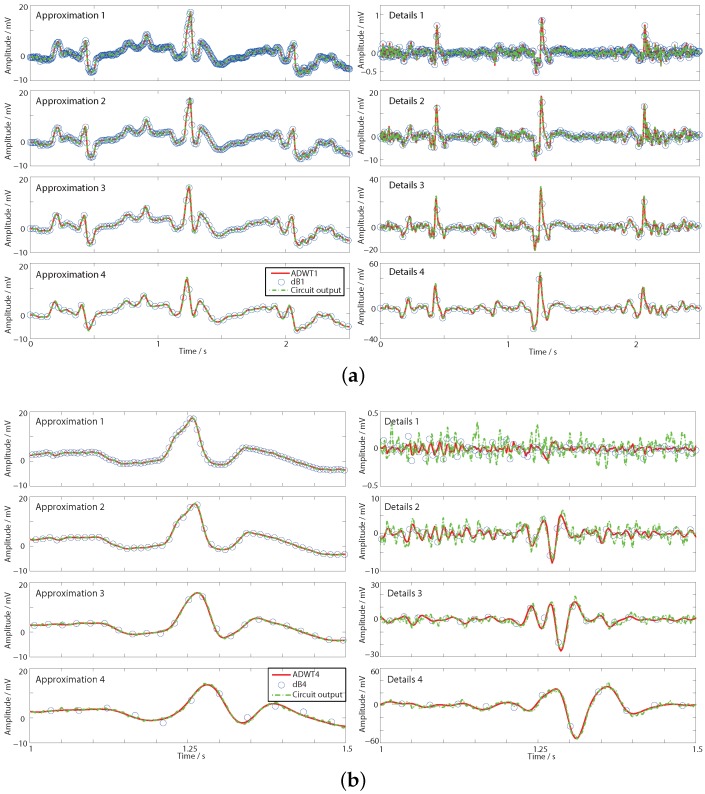
Transient response of the ADWT $g_{m}$C circuits including noise. (**a**) First order ADWT1 for dB1; (**b**) Fourth order ADWT4 for dB4.

The ADWT filters are combined to form a multi-resolution filter bank as shown in Figure 2 by providing scaled copies of the bias current to the different filter stages. Figure 9 shows the transient response of the ADWT circuits performing a multi-resolution analysis using the same noisy ECG input signal as in Figure 6. The Cadence Spectre transient plus noise functionality has been used to incorporate the effects of circuit noise in the presented responses, and the outputs delayed by fixed amounts to show the coefficient alignment. Figure 9 thus demonstrates the successful operation of the filters and shows that useful ADWT information can be generated even in the presence of ECG and circuit noise. As previously, the ideal DWT coefficients all fall on the underlying ADWT line. Requiring only one external bias current, the four level ADWT1 consumes 37 nW of power while the ADWT4 consumes 114 nW, clearly demonstrating the generation of analog domain DWT-like information using nano-Watts of power.

**Table 5:** Performance summary of ADWT filters.

Parameter	A1	D1	A4	D4
CMOS process	0.18 μm, triple well, 6 metal layers, single poly, MIM capacitors available			
Supply voltage	1.3 V			
Bias current	512 pA			
Area	0.12 mm${}^{2}$		0.42 mm${}^{2}$	
Power consumption	16.7 nW	7.2 nW	35.8 nW	29.4 nW
Signal input range	100 mV${}_{pp}$	100 mV${}_{pp}$	100 mV${}_{pp}$	100 mV${}_{pp}$
THD (100 mVpp, 10 or 180 Hz input)	–55.7 dB	–56.5 dB	–52.5 dB	–44.5 dB
Input referred noise (0.16m–3 dB point–1k Hz)	100 μV${}_{RMS}$	70 μV${}_{RMS}$	93 μV${}_{RMS}$	219 μV${}_{RMS}$
Dynamic range	51 dB	54 dB	52 dB	44 dB
Tuning range	2–720 Hz	2–720 Hz	2–720 Hz	2–720 Hz

**Table 6:** Comparison of the ADWT with previously reported digital DWT circuits. Values in italics are either unclear or have been extrapolated from values given in the source paper.

Ref.	Year	Mother Wavelet	Levels	Channels	Process/μm	Analysis Frequency/Hz	V${}_{DD}$	Input Range	Power/W	Area/mm${}^{2}$
[26]	2007	sym4	4	32	0.18	12.5 k	1.3	10 bit	76 μ	0.22
[27]	2007	sym4	4	32	0.13	12.5 k	*–*	10 bit	50 μ	0.69
[28]	2007	dB4 ${}^{a}$	3 ${}^{b}$	1	0.18	*12.5 M*	1.8	16 bit	26 m	0.55
[17]	2010	dB6 ${}^{a}$	7	1	0.35	*69.4 M*	3.3	8 bit	154 m	0.06
[29]	2011	dB3	2	20	0.07	5 k	0.6	8 bit	10 μ	0.21
[30]	2011	Custom	8	*–*	0.18	500	1	9 bit	29 μ	3 ${}^{c}$
[31]	2012	Quadratic spline	3	1	0.35	150	1.8	10 bit	830 n	1.11 ${}^{d}$
[32]	2014	sym4 e	4	32	0.13	13k	1.2	10 bit	800 μ	1.21
[33]	2014	Custom	4	*–*	0.18	125	0.5	9 bit	435 n	*–*${}^{f}$
[34]	2015	dB4 ${}^{a}$	3 ${}^{b}$	1	0.18	180	0.5	16 bit	26 μ	0.53
This work	2015	ADWT1 (dB1)	4	1	0.18	180	1.3	51 dB	37 n	0.48
This work	2015	ADWT4 (dB4)	4	1	0.18	180	1.3	44 dB	114 n	1.68

${}^{a}$ Example used in paper. Circuit can implement arbitrary wavelet bases; ${}^{b}$ Three level Discrete Wavelet Packets Transform circuit which contains 7 DWT blocks; ${}^{c}$ Includes noise removal, reconstruction and beat detection; ${}^{d}$ Includes beat detection and wireless controller; e Includes thresholder and run-length encoder in addition to DWT; ${}^{f}$ Includes noise removal, reconstruction, beat detection and encryption.

Table 6 compares the performance of the ADWT circuits to previously reported 1D DWT circuits when used to perform a multi-resolution analysis. This shows a number of digital DWT circuit implementations which use four level analyses and analysis frequencies in the sub-kHz range, as the ADWT is designed for. All but one of the circuits in Table 6 operates from 1.3 V or below, suitable for powering from a single coin cell battery. Precise comparisons between implementations are difficult because not all papers report performance results for the DWT circuit stage in isolation (many papers report only overall figures for several system blocks). Nevertheless, it is readily seen that the ADWT is highly competitive in terms of power consumption: it has the lowest absolute power consumption, and the lowest power consumption when normalized for the analysis frequency. This is critical for creating ubiquitous wearable sensors where the principal limitation is battery life. Moreover, the key advantage of the ADWT circuit compared to those in Table 6 is that the DWT-like operation and performance is achieved without leaving the analog domain. As noted in the introduction, there is a strong community consensus that the increased use of analog signal processing has an important role in achieving ultra low power consumption signal processing. In addition, time–frequency decompositions are the most common algorithm basis for use in ultra low power, highly embedded signal processing algorithms for wearables [47]. The ADWT combines these opportunities. It allows, for the first time, analog signal processing algorithms to use DWT-like information as their signal processing basis. It also gives the potential for further system power savings in the Analog-to-Digital Conversion and wireless transmission stages as it may not be necessary to sample all of the raw data, instead sampling only a sub-set of DWT levels and coefficients.

## 5. Conclusions

For long operational lifetimes wearable sensor nodes have very limited power budgets and incorporating advanced signal processing within these budgets is a major design challenge. This paper has presented a new low power analog approach for implementing the Discrete Wavelet Transform using under 115 nW of power. The Analog DWT is provided by $g_{m}$C circuits which are readily suitable for on-chip and on-sensor node implementation for low power, low voltage, low frequency applications. Results from the analysis of human ECG and EEG signals have demonstrated that the new analog approach can produce useful DWT-like information, providing the designers of emerging analog signal processing systems with increased flexibility and choices in algorithm design.
